# *Saccharomyces paradoxus* K66 Killer System Evidences Expanded Assortment of Helper and Satellite Viruses

**DOI:** 10.3390/v10100564

**Published:** 2018-10-16

**Authors:** Iglė Vepštaitė-Monstavičė, Juliana Lukša, Aleksandras Konovalovas, Dovilė Ežerskytė, Ramunė Stanevičienė, Živilė Strazdaitė-Žielienė, Saulius Serva, Elena Servienė

**Affiliations:** 1 Laboratory of Genetics, Institute of Botany, Nature Research Centre, LT-08412 Vilnius, Lithuania; igle.vepstaite-monstavice@gamtc.lt (I.V.-M.); juluksa@gmail.com (J.L.); ezerskytedovile@gmail.com (D.E.); ramune.staneviciene@gamtc.lt (R.S.); zivile.strazdaite-zieliene@gamtc.lt (Ž.S.-Ž.); 2 Department of Biochemistry and Molecular Biology, Institute of Biosciences, Vilnius University, LT-10257 Vilnius, Lithuania; aleksandras.konovalovas@gf.vu.lt; 3 Department of Chemistry and Bioengineering, Vilnius Gediminas Technical University, LT-10223 Vilnius, Lithuania

**Keywords:** *Saccharomyces paradoxus*, *Totiviridae*, dsRNA virus, killer system

## Abstract

The *Saccharomycetaceae* yeast family recently became recognized for expanding of the repertoire of different dsRNA-based viruses, highlighting the need for understanding of their cross-dependence. We isolated the *Saccharomyces paradoxus* AML-15-66 killer strain from spontaneous fermentation of serviceberries and identified helper and satellite viruses of the family *Totiviridae*, which are responsible for the killing phenotype. The corresponding full dsRNA genomes of viruses have been cloned and sequenced. Sequence analysis of SpV-LA-66 identified it to be most similar to *S. paradoxus* LA-28 type viruses, while SpV-M66 was mostly similar to the SpV-M21 virus. Sequence and functional analysis revealed significant differences between the K66 and the K28 toxins. The structural organization of the K66 protein resembled those of the K1/K2 type toxins. The AML-15-66 strain possesses the most expressed killing property towards the K28 toxin-producing strain. A genetic screen performed on *S. cerevisiae* YKO library strains revealed 125 gene products important for the functioning of the *S. paradoxus* K66 toxin, with 85% of the discovered modulators shared with *S. cerevisiae* K2 or K1 toxins. Investigation of the K66 protein binding to cells and different polysaccharides implies the β-1,6 glucans to be the primary receptors of *S. paradoxus* K66 toxin. For the first time, we demonstrated the coherent habitation of different types of helper and satellite viruses in a wild-type *S. paradoxus* strain.

## 1. Introduction

Yeasts constitute a large group of microorganisms characterized by the ability to grow and survive in stressful conditions and to colonize a wide range of environmental ecosystems [1]. The secretion of yeast killer toxins confers a competitive edge to the producer strain by excluding other yeasts from shared habitat without direct cell-to-cell contact [2]. Rather than providing immediate advantages for the respective host, killer toxin-immunity systems also have to be considered as important players in the autoselection system [3]. Recently, the mutual incompatibility of double-stranded RNA virus-based killer systems and the RNA interference mechanism in yeast has been demonstrated [4].

Double-stranded RNA-based killer systems have been described in different yeast species, such as *Saccharomyces cerevisiae*, *S. paradoxus*, *S. uvarum*, *Ustilago maydis*, *Zygosaccharomyces bailii*, *Hanseniaspora uvarum*, and *Torulaspora delbrueckii* [5,6,7]. The mycoviruses of the *Totiviridae* family are incapsulated into virus-like particles (VLPs) and stably persist in the host cell without causing cell lysis; they are transmitted by vegetative cell division or through sexual fusion [5,8,9,10]. Most killer toxins are encoded by dsRNA viruses called M satellites, which depend for their propagation and maintenance on an L-A helper virus [8,11]. Among the representatives of the genus *Saccharomyces*, the killer phenomenon has been studied most extensively in *S. cerevisiae*, where four different viral-originated killer toxins (K1, K2, K28, and Klus) have been described [12,13,14,15]. For effective functioning of the killer system, well organized communication between the functionally distinct ScV-LA and ScV-M viruses is required. The L-A virus has a 4.6 kb segment that encodes the major structural capsid protein Gag, encapsulating either L or M virus dsRNA in icosahedral structures, and the Gag-Pol fusion protein responsible for replication and encapsidation [11,16,17]. There are several variants of L-A (ScV-LA-1, ScV-LA-2, ScV-LA-28 and ScV-LA-lus), with an average of 74% identity in nucleotide sequences, associated with different M viruses and displaying distinct phenotypic properties [14,18]. Four different *S. cerevisiae* M dsRNA viruses have been described (ScV-M1, ScV-M2, ScV-M28 and ScV-Mlus) so far [15]. Certain relationships between L-A and M viruses have been observed (LA-1 and M1, LA-2 and M2, LA-lus and Mlus, LA-28 and M28) [14,18,19] and the possible role of the toxin-producing M viruses in selecting the L-A variants to support them has been proposed [19]. A certain heterogeneity in L-A itself has also been reported with functional phenotypic variants that exhibited differences in maintaining the K1 and K2 phenotypes with the involvement of MKT genes [20]. The observed exceptions of specificity were essentially limited to laboratory strains or hybrids, as well as strains featuring significantly elevated amounts of L-A dsRNA or proteins encoded by this virus [19,20]. The association of distinct L-As with different M viruses suggests their co-evolution, leading to the propensity of a particular L-A virus to maintain a certain type of M virus [14,19].

The M viral genome is 1.6–2.4 kb in size and encodes a specific preprotoxin, which is subsequently processed into a mature protein (K1, K2, K28, and Klus). The M dsRNA viruses show no sequence homology to each other, though the organization of their genomes is strikingly similar. The positive strand contains an open reading frame (ORF) in the 5‘-terminal region that encodes for the toxin precursor, followed by a unique internal AU-rich region, and a 3‘-terminal non-coding region of variable length possessing *cis* signals for encapsidation and replication by the viral RNA polymerase [6]. Different killer toxins are secreted glycoproteins lacking amino acid sequence conservation and adopting diverse cell killing mechanisms. The proposed mechanism of the action for the K1 and K2 toxins is a two-step process, whereby the killer protein first binds to the primary cell wall receptor-β-1,6-glucan [21,22], then, at the second step, the toxins approach a plasma membrane receptor and form lethal cation-selective ion channels [15,23,24,25]. In contrast, the K28 toxin binds to α-1,3-mannoproteins positioned in the cell wall, then interacts with a plasma membrane receptor Erd2 and enters the cell by endocytosis. In the cell, the K28 toxin travels to the nucleus by retrograde passage and blocks DNA synthesis causing G1/S cell cycle arrest [26,27]. The lethal mechanism of the Klus toxin is yet to be uncovered [14,28].

Many host genes affect the maintenance of *S. cerevisiae* L-A and M viruses [11], as well as the performance of viral killer toxins [29,30,31]. The SKI family genes block expression of the non-polyadenylated viral mRNA [32]. Species-specific virus restriction factor Xrn1p (encoded by SKI1 gene) appears to co-evolve with totiviruses to control viral propagation in *Saccharomyces* yeasts [33]. MAK family gene products are necessary for L-A and M propagation [12,34,35]. The exact function and interplay of these genes in virus replication and maintenance is not fully understood [36,37]. On the other hand, the presence of dsRNA viruses impacts the expression of numerous host genes [38,39], many of them tightly integrated into cellular metabolism. The phylogenetic analysis uncovers that at least some mycoviruses co-evolve with their hosts, suggesting a close interaction between participants [40,41]. Viruses and their hosts exist in a constant state of genetic conflict, where an advantage for one party is often a disadvantage for the other [33]. The stable persistence of L-A virus in some 20% of wild *S. cerevisiae* [42] cells suggests a generally detrimental impact to the host, probably because of consumption of energy and material resources [11]. In addition, M dsRNAs are rather rare in wild strains [42,43].

*Saccharomyces paradoxus* is the closest relative of the domesticated yeast *S. cerevisiae*, mainly found in the wild [6]. Killer toxins of *S. paradoxus* have been considered as chromosome-coded for a long time and it is only relatively recently that dsRNA viruses (L and M) in such yeast have been discovered [44]. The sizes of L and M dsRNAs genomes are similar to those of *S. cerevisiae* strains. The degree of nucleotide variation in different types of L-A viruses ranged from 73% to 90%, depending on the geographical location of *S. paradoxus* strains [6,9]. The essential features of *S. cerevisiae* L-A viruses-frameshift region and encapsidation signal-remain conserved in all *S. paradoxus* L-A variants investigated thus far. The encoded Gag-Pol proteins demonstrate 85% to 98% amino acid identity. At least five different *S. paradoxus* killer toxin-producing viruses (SpV-M21, SpV-M28, SpV-M45, SpV-M62, and SpV-M74) have been identified. They encode toxins differing in sequences from any of those previously known, while structural-functional characterization has not been accomplished yet [6].

In this study, we performed cloning and structure-functional analysis of the *S. paradoxus* SpV-LA-66 and SpV-M66 viruses, recently isolated from the natural environment. Comparison of the SpV-LA-66 sequence and phylogenetic analysis demonstrated its close relationship to SpV-LA-28 and SpV-LA-21 viruses. The SpV-M66 virus-encoded preprotoxin sequence analysis allowed prediction of the structural elements of the active K66 killer protein. The *S. paradoxus* K66 toxin was isolated and the essential parameters of protein activity were investigated. For the first time, we have identified genetic factors, involved in the functioning of the *S. paradoxus* virus-originated K66 toxin, and those important for the susceptibility of the target cell. The gene products, connected to cell wall organization and biogenesis, as well as involved in the regulation of response to osmotic stress, were demonstrated as significantly enriched. By performing in vivo and in vitro toxin binding assays, we demonstrated that β-1,6 glucans could play the role of primary receptors for the *S. paradoxus* K66 toxin. We concluded that SpV-LA-66 and SpV-M66 represent a previously undescribed combination of helper and satellite viruses in the wild-type *S. paradoxus* AML-15-66 strain.

## 2. Materials and Methods

### 2.1. Strains and Media

The killer strain employed in this study (*Saccharomyces paradoxus* AML-15-66) was originally isolated from spontaneous fermentation of serviceberries (*Amelanchier ovalis* Medik.). The following yeast strains were used for the killer assay: *S. cerevisiae* α’1 (*MATα leu2-2* (*KIL-0*)) [45], M437 (*wt, HM/HM* (*KIL-K2*)) [46], K7 (*MATα arg9* (*KIL-K1*)) [47], MS300 (*MATα leu2 ura 3-52* (*KIL-K28*)) [13], SRB-15-4 (wt, HM/HM (KIL-Klus) (laboratory collection), BY4741 (*MATa; his3 D1*; *leu2∆0*; *met15∆0*; *ura3∆0* (*KIL-0*)) (Thermo Scientific Molecular Biology, Lafayette, CO, USA), and *S. paradoxus* T21.4 strain (kindly provided by Dr. G. Liti, Université Côte d’Azur, CNRS, INSERM, IRCAN, Nice, France). The curing of yeast strains from dsRNA viruses was accomplished as described in [39].

For identification of yeast, the regions between the 18S rRNA and 28S rRNA genes containing two non-coding spacers (ITS-A and ITS-B) separated by the 5.8S rRNA gene were PCR-amplified using ITS1 (5′-TCCGTAGGTGAACCTGCGG-3′) and ITS4 (5′-TCCTCCGCTTATTGATATGC-3′) primers [48], and sequenced at Base Clear (Leiden, ZH, The Netherlands). The obtained sequences were compared with those found in the FASTA network service of the EMBL-EBI database (http://www.ebi.ac.uk/Tools/sss/fasta/nucleotide.html). Screening for genetic factors modulating the K66 toxin activity was performed with a *S. cerevisiae* single ORFs deletion strains (BY4741 background, *MATa*; *his3 D1*; *leu2∆0*; *met15∆0*; *ura3∆0*) (Thermo Scientific Molecular Biology, Lafayette, CO, USA).

Yeast strains were grown in standard YEPD medium (1% yeast extract, 2% peptone, 2% dextrose, 2% agar). For the killing assay, MBA medium (0.5% yeast extract, 0.5% peptone, 2% dextrose) was used, adjusted to appropriate pH 3.2–6.0 with the 75 mM phosphate-citrate buffer and supplemented with 0.002% methylene blue dye. For toxin preparation, liquid synthetic medium SC (2% dextrose, 0.2% K_2_HPO_4_, 0.1% MgSO_4_×7H_2_O, 0.1% (NH_4_)_2_SO_4_, 1.29% citric acid, 2.76% Na_2_HPO_4_×12H_2_O) was used, containing 5% glycerol, adjusted to appropriate pH 3.2–6.0.

### 2.2. Assay for Killing/Resistance Phenotypes

For detection of killing phenotype, the tested *S. paradoxus* strain was spotted on the MBA agar plates seeded with a lawn of the sensitive *S. cerevisiae* strain BY4741 (2 × 10^6^ cells/plate) or strains of different yeast species. After incubation of the plates at 25 °C for 3 days, clear zones of growth inhibition surrounding the killer cells were evaluated and interpreted as a killer activity.

The sensitivity/resistance tests were performed by spotting *S. cerevisiae* killer strains onto the MBA plates with an overlay of the *S. paradoxus* strain AML-15-66. The absence of lysis zones indicates a resistant phenotype, while non-growth zones around the different types of killer toxins producing colonies were attributed to the sensitive phenotype [49].

### 2.3. Viral dsRNA Isolation From Yeast

Total extraction of nucleic acids from yeast was based on the previously described method [50] with modifications. *S. paradoxus* culture was grown in YEPD media overnight at 30 °C. Cells were collected by centrifugation for 5 min at 5000× *g* at 20 °C and washed with 1/10 part of the starting volume of the culture media supplemented by 50 mM EDTA. Cells were collected and re-suspended in 1/10 part of the starting volume of TB buffer (50 mM Tris-HCl pH 9.3; 1% β-mercaptoethanol) and incubated for 15 min at the room temperature. Cells were pelleted and re-suspended in 2/10 part of the starting volume of TES buffer (10 mM Tris-HCl pH 8.0; 100 mM NaCl; 10 mM EDTA; 0.2% (*w/v*) SDS). Subsequently, an equal volume of phenol (pH 5.2) preheated to 80 °C was added, and the suspension was vigorously shaken for 45 min at room temperature. Afterwards, an equal volume of chloroform was added and mixed thoroughly. The mix was subjected to centrifugation at 18,000× *g* for 45 min at 4 °C and separated by pipetting. Nucleic acids were pelleted from an aqueous fraction by adding 1 volume of isopropanol supplemented with 1/10 volume of 3 M sodium acetate (pH 5.2) and subjected to centrifugation at 18,000× *g* for 10 min at 4 °C. The resulting pellets were washed with 75% ethanol and dissolved in DEPC-treated water. Re-suspended nucleic acids were separated in 1% agarose gels and visualized by staining with ethidium bromide.

For double-stranded RNA (dsRNA) preparation, an isolated 700 µg of the total nucleic acid fraction was incubated in 2.8 M LiCl overnight at 4 °C [51]. The single-stranded nucleic acids were removed by centrifugation at 18,000× *g* for 45 min at 4 °C. The aqueous phase was substituted with 1/10 volume of 3 M NaCl and 1 volume of isopropanol, dsRNA pelleted by centrifugation at 18,000× *g* for 10 min at room temperature, washed with 75% ethanol and dissolved in DEPC treated water. The resulting L and M dsRNAs were visualized by gel electrophoresis and gel-purified using GeneJet Gel Extraction Kit (Thermo Fisher Scientific, Vilnius, Lithuania).

### 2.4. cDNA Synthesis, Amplification, and Cloning

Viral dsRNA cDNA synthesis and amplification were performed as described [52], with modifications. PC3-T7 loop primer (5′-GGATCCCGGGAATTCGGTAATACGACTCACTATATTTTTATAGTGAGTCGTATTA-3′) was ligated to gel-extracted dsRNA following the primer:dsRNA molar ratio of 250:1. The ligation reaction was performed by T4 RNA ligase (Thermo Fisher Scientific, Vilnius, Lithuania) in the supplier’s buffer with additional adding of up to 20% PEG-6000, 10% DMSO, 0.01% BSA and 20U RiboLock RNase Inhibitor (Thermo Fisher Scientific, Vilnius, Lithuania) overnight at 37 °C. DsRNA with ligated primers was purified using a GeneJet PCR Purification Kit (Thermo Fisher Scientific, Vilnius, Lithuania), before cDNA synthesis step was denatured by adding of DMSO to a final concentration of 15% (*v/v*), heating at 95 °C for 2 min, and immediately transferred onto the ice for 5 min. Prepared dsRNA was reverse-transcribed using Maxima Reverse Transcriptase (Thermo Fisher Scientific, Vilnius, Lithuania). Alkaline hydrolysis of residual RNA was performed and cDNA strands re-annealed at 65 °C for at least 90 min followed by gradual cooling to 4 °C for 2 h The cDNA was amplified using Phusion High-Fidelity DNA Polymerase (Thermo Fisher Scientific, Vilnius, Lithuania) with PC2 primer (5′-CCGAATTCCCGGGATCC-3′) by using the manufacturer’s recommended cycling conditions, with an initial step carried at 72 °C for 2 min added. PCR products were cloned into the pUC19 vector (Thermo Fisher Scientific, Vilnius, Lithuania), sequenced at Base Clear (Leiden, ZH, The Netherlands) and the obtained sequences blasted against known sequences in the NCBI database.

### 2.5. Sequence Analysis

A maximum likelihood phylogenetic tree was constructed using the IQ-Tree v1.6.3 [53] with automatic selection of best-fit amino acid substitution and site heterogeneity models. LG + R3 proved to be the best-fit model. Edge support was estimated with bootstrap test (1000 replicates). Phylogenetic tree visualization was performed using Fig-Tree v1.4.3 program (http://tree.bio.ed.ac.uk/software/figtree/). Phobius server http://phobius.sbc.su.se/ [54] was employed for transmembrane topology identification. Sites of N-glycosylation in the protein sequences were identified using NetNGlyc web server (http://www.cbs.dtu.dk/services/NetNGlyc/). Conservative domains of the protein were determined using Pfam database [55]. DIANNA server (http://clavius.bc.edu/~clotelab/DiANNA) [56] was employed for disulfide bond connectivity prediction.

### 2.6. Analysis of the Partially Purified K66 Toxin for Thermal and pH Activity

*S. paradoxus* strain AML-15-66 was grown in synthetic SC-medium at various pH in the range of 3.2–6.0 for 4 to 6 days at 18 °C until reaching comparable cell density (OD600: 0.6–0.8). Yeast cells were separated by centrifugation at 3000× *g* for 10 min and filtration of supernatant through a 0.22 μm sterile PVDF membrane (Millipore, Bedford, MA, USA). The supernatant was then filtered using pressure-based Amicon system (membrane MWCO 10 kDa, Sigma-Aldrich, St. Louis, MO, USA) and serial centrifugations followed through Amicon ultra centrifugal filters with different cut-offs (10 and 30 kDa). The preparation of partially purified K66 toxin was used for assessment of optimal toxin activity and screening for modulators.

To determine K66 toxin activity at different pH values, MBA plates adjusted to pH values between 3.2 and 6 were seeded with the sensitive *S. cerevisiae* strain α’1 (2 × 10^6^ cells/plate) and incubated at 25 °C in the presence of aliquots of the toxin (100 µL) extracted from killer protein-producing strain grown at appropriate pH and concentrated 100-fold. The inhibition zones were determined on triplicate plates after 2–5 days of incubation and the mean of remaining toxin activity was expressed in percent. Toxin preparation obtained at pH 4.8 was used for temperature activity measurement following the method described in [57].

### 2.7. Screening for Modulators of K66 Activity and Bioinformatic Analysis

The sensitivity was tested by either depositing 100 mL of concentrated 100-fold K66 toxin into 10 mm diameter “punched-wells” in the agar plate or spotting K66-producing cells onto the MBA medium overlaid with the yeast strain of interest (2 × 10^6^ cells/plate). Plates were incubated for 2 days at 25 °C, and the diameter of the lysis zones measured and compared with those formed on BY4741 overlay. The screening was repeated 3 times.

The GO-term analysis was performed using the BiNGO 3.0.3 plug-in embedded into the Cytoscape 3.6.1. platform [58]. Significance *p* values were calculated with the hypergeometric test, using the Benjamini and Hochberg false discovery rate (FDR) correction for the enrichment of each GO term. Fold enrichment (F.E.) was determined by dividing the frequency of specific gene cluster to the total frequency for each GO term.

Network diagrams were generated using STRING web resource (version 10.5, http://string-db.org) [59]. Our created network uses the “confidence view” option of the program, where stronger associations are represented by thicker lines. The experiments-based active prediction method was used, and the medium confidence score (0.400) was utilized.

### 2.8. Evaluation of K66 Toxin Binding to the Yeast Mutants and Different Polysaccharides

*S. cerevisiae* BY4741 as control and mutant strains from *S. cerevisiae* deletion library were cultivated at 30 °C in YEPD medium overnight. 2 × 10^6^ cells were sedimented by centrifugation at 3000× *g* for 10 min and washed with 1 mL SC medium, pH 4.8 and incubated with 500 µL of 100-fold concentrated K66 toxin at 4 °C for 1 h. The supernatant was collected by centrifugation 1 min 10,000× *g* and tested by the well-test. After 2 days incubation at 25 °C the diameter of lysis zones was measured.

For analysis of K66 binding to different polysaccharides, 9 mg of chitin, laminarin, pullulan, or pustulan were mixed with 100 µL of 100-fold concentrated K66 toxin. Samples were incubated for 1 h at 25 °C. After centrifugation (1 min 10,000× *g*), 100 µL of supernatant was transferred into the wells on MBA medium (pH 4.8) containing α’1 cells. Lysis zones were analyzed after 2 days of incubation at 25 °C.

### 2.9. GenBank Accession Numbers

The SpV-L-A66 and SpV-M66 cDNA nucleotide sequences appear in NCBI/GenBank under GenBank accession no. MH784501 and MH784500, respectively.

## 3. Results

### 3.1. Characterization of the S. paradoxus Killer Strain

The yeast strain AML-15-66 was isolated from spontaneous fermentation of serviceberries. Based on the ITS region sequencing data and RFLP-PCR profiles, the strain was identified as *S. paradoxus* (Figure S1). We observed that AML-15-66 exhibits killing activity against *S. cerevisiae* non-killer and different types of killer virus-possessing strains at pH ranging from 3.6 to 5.6 (Table 1).

The strongest killing activity was determined at pH 4.4–4.8 against non-killer *S. cerevisiae* strains α‘1 and BY4741. The killing phenotype against various types of killer toxin-producing strains was weaker, compared to that of the killer-free strains. *S. paradoxus* AML-15-66 strain demonstrated the lowest activity against *S. cerevisiae* M437 cells maintaining ScV-M2 virus (Table 1) and was not active against tested *Pichia*, *Hanseniaspora*, *Candida*, and *Torulaspora* spp. *S. paradoxus* AML-15-66 strain was found to be resistant to the action of *S. cerevisiae* K1, K28, and Klus mycotoxins as well as to the K21 toxin, produced by *S. paradoxus* T21.4 strain, while susceptible to the action of *S. cerevisiae* K2 toxin produced by strain M437 only (Figure S2A).

### 3.2. Double-Stranded RNA Viruses from the S. Paradoxus Strain

**Figure 1:**
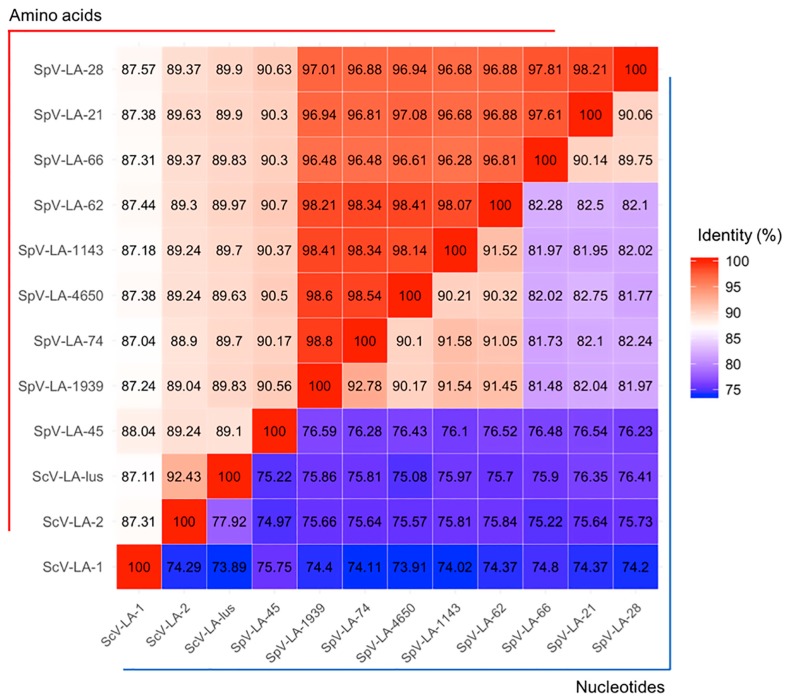
The similarities of *S. cerevisiae* and *S. paradoxus* dsRNA L-A virus-encoded Gag-pol proteins. ORFs coding for the Gag-pol proteins were compared with corresponding fragments of *S. cerevisiae* and *S. paradoxus* dsRNA sequences, namely: GenBank entry J04692 for the ScV-LA-1 virus, KC677754 for ScV-LA-2, and JN819511 for ScV-LA-lus, KU845301 for SpV-LA-28 (formerly attributed to *S. cerevisiae*), KY489962 for SpV-LA-21, KY489963 for SpV-LA-45, KY489964 for SpV-LA-74, KY489965 for SpV-LA-4650, KY489966 for SpV-LA-1939, KY489967 for SpV-LA-1143, KY489968 for SpV-LA-62. Identity at nucleotide level is represented in the lower right triangle, amino acid level–in the upper left triangle, framed by corresponding blue and red lines.

To delve into the nature of the killing phenotype, we extracted dsRNAs from AML-15-66 and performed electrophoretic analysis (Figure S3). The size of observed L and M dsRNAs was compared to that of the dsRNAs isolated from reference strains of *S. cerevisiae* K7 (LA-1, M1), M437 (LA-lus, M2), MS300 (LA-28, M28), and SRB-15-4 (LA-lus, Mlus). The size of the L fraction was about 4.6 kb and thus highly similar to all L dsRNAs, while M dsRNA was about 1.6 kb and thus close to M2 dsRNA. We named these viruses SpV-LA-66 and SpV-M66, respectively. Purified dsRNA of the SpV-LA-66 virus was used as a substrate for primer ligation, subsequent reverse transcription, and cDNA amplification. In total, the genome of the SpV-LA-66 virus was found to possess 4580 nucleotides. Like other known L-A viruses, SpV-LA-66 genome features two overlapping open reading frames, which encode capsid protein Gag and RNA dependent RNA polymerase Gag-pol, formed by ribosomal frameshift. All features inherent for L-A viruses, such as conservative frameshift region, packing and replication signals, and catalytic histidine residue required for cap-snatching were present in the SpV-LA-66 genome sequence. Tentative ORFs coding for the Gag-pol and Gag proteins were compared with corresponding fragments of *S. cerevisiae* and *S. paradoxus* dsRNA sequences. At the nucleotide level, all entries display 74 to 92% identity (Figure 1).

**Figure 2:**
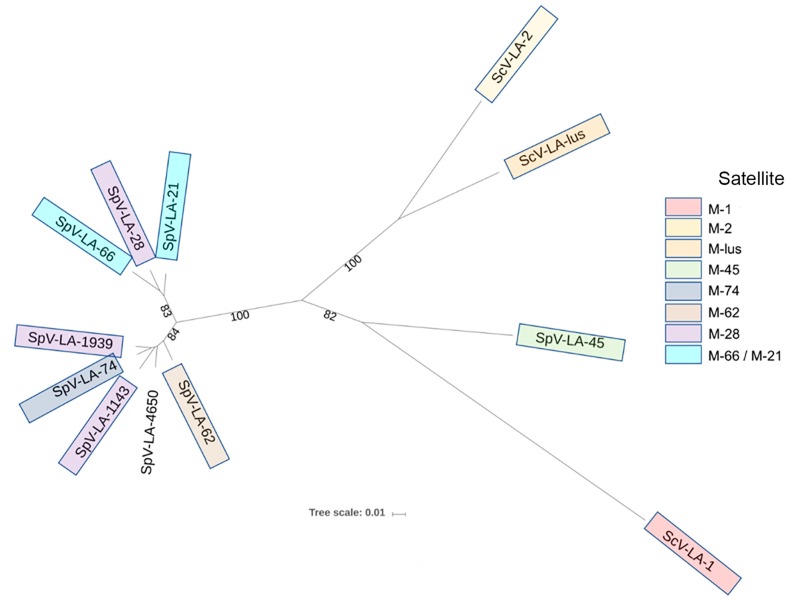
The phylogenetic tree of dsRNA-encoded Gag-pol proteins from *S. cerevisiae* and *S. paradoxus* yeasts.

Similarity at the amino acid level is higher: coat proteins (Gag) are 88 to 99% homologous (Figure S4), while RNA polymerases (Gag-pol) are 87 to 98% homologous (Figure 1). Proteins originating from SpV-LA-66, SpV-LA-28 (initially reported as ScV-LA-28, origin recently updated by [6]) and SpV-LA-21 are the most closely related and comprise a separate cluster in relation to other L-A viruses (Figure 2). The remaining L-A viruses from *S. paradoxus* comprise another cluster. Altogether, *S. paradoxus* L-A viruses are significantly more homogenous than those from *S. cerevisiae*.

**Figure 3:**
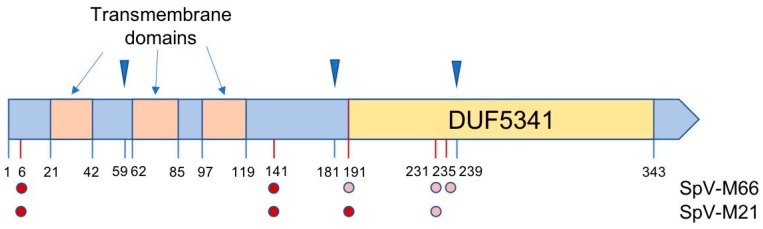
Features of the protein coded by SpV-M66. Blue triangles above the picture mark Kex2 sites. Predicted transmembrane domains and DUF5341 conservative Pfam family domain are marked within the picture. Dots below the picture mark putative glycosylation sites for K66 and K21 proteins, color intensity corresponds to the value of reliability index of the given position. Feature-linked positions of amino acids are indicated.

In the same AML-15-66 strain, we discovered and cloned a 1553 bp long M dsRNA, named as SpV-M66. Sequence analysis of M66 satellite shows 86% identity in nucleotides to *S. paradoxus* SpV-M21 virus (GenBank MF358732) (Figure S5). The SpV-M66 genome consists of 5′-end 4 bp non-translating region, single ORF of 1,038 bp, which has 92% aa identity to K21 killer preprotoxin (GenBank ATN38270), about 60 bp polyA region and 330 bp non-translating region located at 3′-end (Figure S6). We expressed the complete ORF sequence from SpV-M66 in *S. cerevisiae* BY4741 strain and confirmed that the encoded protein confers the host with the killer activity (Figure S2B). Sequence analysis of preprotoxin revealed three potential recognition sites for Kex2 protease ending at amino acids Arg59, Arg181, and Arg239 (Figure 3, Figure S5); prediction of disulfide bridge formation sites remains ambiguous. Up to five putative protein N-glycosylation sites were found in the sequence of the K66 protein, four of them overlapping with those for K21. The amino acid at 141 position has the highest probability to be modified. A hydrophobicity profile reveals three transmembrane domains (from 21 to 42 aa, 62 to 85 aa, and 97 to 119 aa) in the K66 protein (Figure 3). In the C-proximal part of K66 precursor, a conservative Pfam family domain DUF5341 of presumably unknown function has been identified.

### 3.3. Effect of pH and Temperature on the Action of the S. Paradoxus K66 Toxin

**Figure 4:**
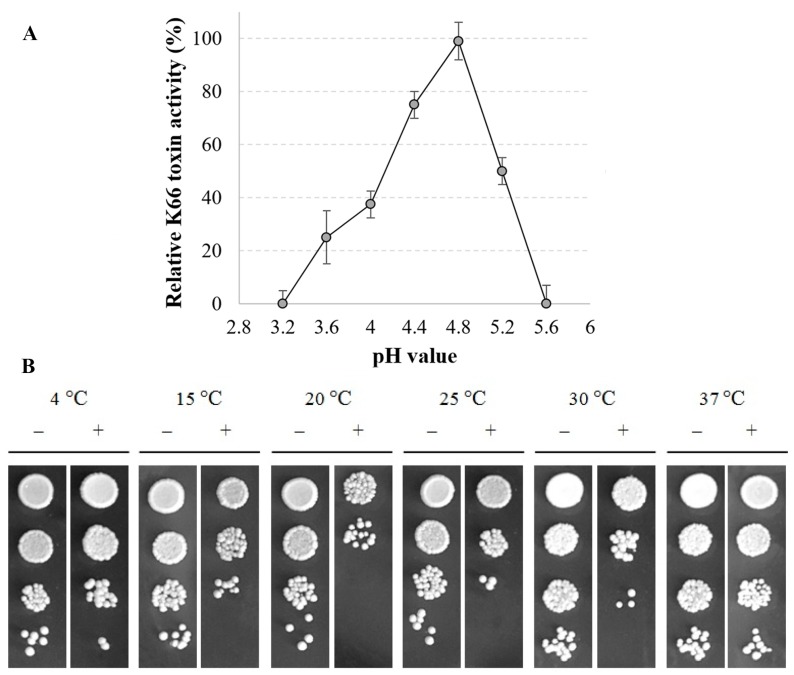
Impact of pH and temperature on K66 toxin functionality. (**A**) Sensitive *S. cerevisiae* strain a’1 was seeded into MBA medium (2 × 10^6^ cells/plate, adjusted to pH values between 3.2 and 6) and 100 µL of the concentrated K66 toxin poured into 10 mm wide wells, cut in the agar layer. Plates were incubated for 2 days at 25 °C, non-growth zones around the wells measured and expressed as mean of three independent experiments in percent ± SD. (**B**) Sensitive yeast a’1 cells (5 × 10^5^ cells) were mixed with 500 µL of 100-fold concentrated K66 toxin or the same volume of heat-inactivated K66 toxin and incubated for 24 h at different temperatures. Yeast cells were then serially diluted and spotted onto YPD-agar plates following for 2 days incubation at 25 °C.

The activity of partially purified *S. paradoxus* K66 viral protein was assayed on a lawn of *S. cerevisiae* strain α’1 with adjusted pH value from 3.2 to 6. K66 toxin exhibits killing activity in a narrow pH range between 3.6 and 5.2, with an activity peak at pH 4.4–4.8 (Figure 4A).

More acidic pH values of 4.0–3.6 result in a reduction of toxin activity up to 38% and 25% respectively, and more basic than optimal pH 5.2 results in about 50% of toxin activity remained (Figure 4A). By analyzing yeast cells that survived the treatment by K66 protein at different temperatures (from 4 °C to 37 °C), we found that extracted viral protein is active at temperatures between 15 °C and 30 °C, with optimal temperature of 20 °C (Figure 4B).

### 3.4. Genetic Factors Modulating the Functionality of the Viral K66 Toxin

To determine the genetic factors important for the action of *S. paradoxus* K66 toxin and involved in the formation of cellular resistance to the viral agent, we screened 526 *S. cerevisiae* single-gene deletion mutants, previously demonstrated to alter the functioning of *S. cerevisiae* K1, K2, or K28 toxins [29,30,31].

**Figure 5:**
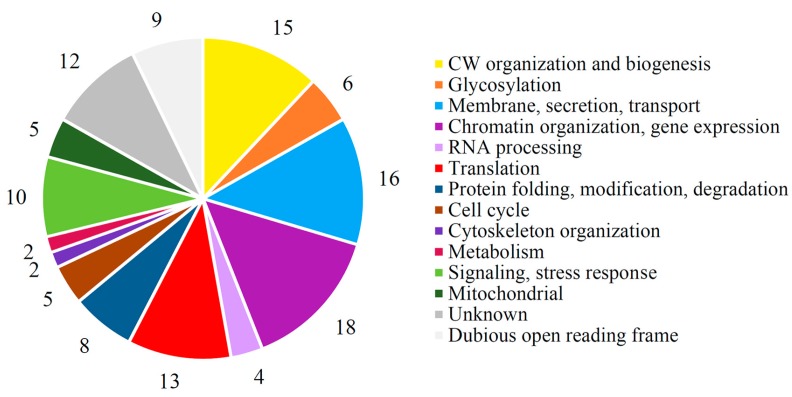
Distribution of cellular processes and cellular components involved in the action of K66 toxin. The number of genes identified in each class is indicated.

We identified 125 *S. cerevisiae* YKO library mutants demonstrating different degrees of phenotypic response to the K66 toxin (Table S1), of which 73 were more resistant than the control strain BY4741 and 52 more sensitive to the toxin treatment. We manually annotated groups of all identified mutants resistant or susceptible to K66 toxin. The largest groups contain genes associated with cell wall organization and biogenesis (15), membrane formation/secretion/transport (16), chromatin organization/gene expression (18), and translation (13) (Table S1, Figure 5). Deletions of 19 genes were common in all four screens performed and cause resistance/sensitivity alterations in the cells, not depending on the toxin type. In 13 mutants, different responses to K66 and some *S. cerevisiae* toxins were recorded (Table S1, Figure S2C). 85% of modulators identified in *S. paradoxus* K66 screen (106 gene products) were also identified in screens of *S. cerevisiae* K2 or K1 toxins.

The GO-term analysis (“biological process”) reveals a statistically significant enrichment in genes involved in cell wall organization and biogenesis (F.E. (fold enrichment) of 4.1, *p* < 5.4 × 10^−6^), response to osmotic stress (F.E. of 5.6, *p* < 2.7 × 10^−3^), and signaling pathway (F.E. of 3.3, *p* < 1.3 × 10^−3^) (Table S2).

**Figure 6:**
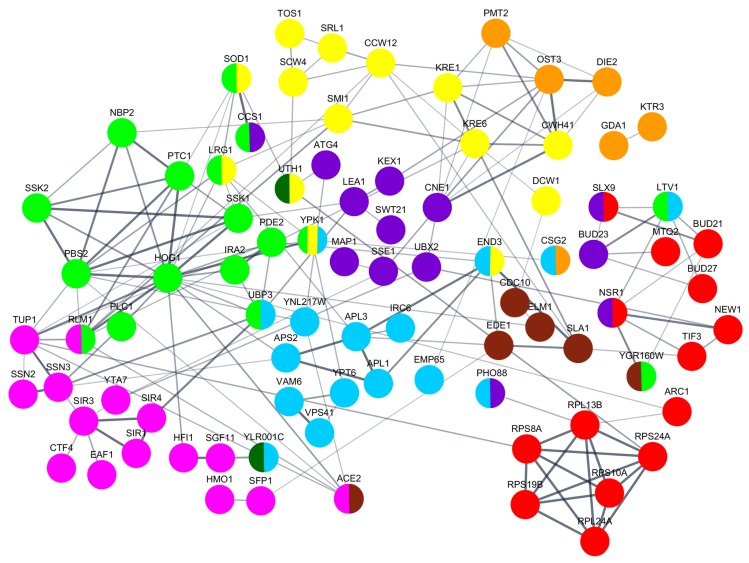
Interconnections of gene products involved in the modulation of susceptibility to K66 toxin. An integrated functional interaction network is obtained from STRING database. Subnetworks of proteins associated with ribosomes/translation (red), signaling and stress response (green), chromatin organization and gene expression (purple), glycosylation (orange), CW organization/biogenesis (yellow), cell cycle (brown), membrane and transport (light blue), RNA and protein modification (dark blue), and mitochondrial (dark green) are represented.

Based on published high-throughput datasets, we built a protein-interconnection network and documented that the majority of all identified genetic factors were a part of one main functional cluster, at medium confidence level (0.4) (Figure 6). Most of the observed proteins are involved in stress response and signaling processes, cell wall organization and biogenesis, belong to ribosomal components or translation machinery, and are connected to membranes or protein transport.

### 3.5. Targeting of the Viral K66 Killer Protein to the Cell Wall

**Figure 7:**
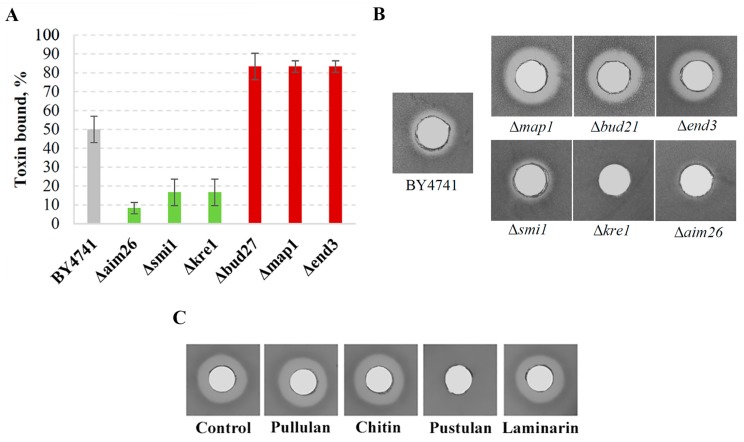
K66 toxin binding to yeast mutants with altered levels of β-glucans and different polysaccharides. (**A**) Cells of different yeast mutants (2 × 10^6^ each) were incubated with 500 µL of concentrated K66 toxin, the remaining toxin activity was measured by the well assay. After incubation with the indicated yeast strain, the unbound K66 toxin is able to kill sensitive tester strain α’1, seeded in the MBA plates. The size of the formed lysis zones was converted to the relative toxin activity and subtracted from the total activity to calculate the binding efficiency. The data are averages ± standard deviations (SD) (n = 3). (**B**) Responses of deletion strains to the action of K66 toxin were measured in the well assay using BY4741 strain in a lawn. (**C**) Nine milligrams of each polysaccharide (chitin, laminarin, pullulan, or pustulan) in 100 µL of concentrated K66 toxin preparation was incubated for 1 h at 25 °C and residual toxin activity was analyzed in the well assay using sensitive α’1 strain in the lawn.

To correlate the K66 toxin binding with the cell wall composition, we investigated the binding properties of the viral protein to mutant cells with altered levels of β-1,3 and β-1,6 glucans [21,29]. The level of glucan content was based on that reported in [21,29]. During this study, we determined that mutants with decreased levels of β-1,6 glucans *Δaim26*, *Δsmi1*, and *Δkre1* bind from 35% to 42% less of the K66 toxin molecules than control cells (BY4741) (Figure 7A). The killing activity of K66 is independent of β-1,3 glucan concentration in the cell wall (β-1,3 glucan amount in *Δaim26* is as in *wt*, *Δsmi1*—50% decreased and in *Δkre1*—10% increased). When mutant cells have increased amount of β-1,6-linkages at the cell wall, as in *Δbud27*, *Δmap1*, and *Δend3* mutants, K66 binding is boosted at about 30% over the control cells level. The defects in cell wall structure resulted in a major impact on the efficiency of K66 toxin binding to the cells, as in the case of K2 and K1 killer toxins [21,29,31,60]. We observed a good correlation between the genes whose deletion led to decreased K66 toxin binding and increased resistance to the toxin. Similarly, genes whose deletion led to increased toxin binding correlated with increased sensitivity towards the K66 toxin (Figure 7B).

To confirm the in vivo data on the importance of β-1,6 glucans as a binding target of K66, the ability of toxin to directly complex the polysaccharides bearing different glucan linkages was evaluated. After incubation with either laminarin (consisting of β-1,3 and β-1,6 linkages), pustulan (β-1,6 linkage), pullulan (α-1,4 and α-1,6 linkages), or chitin (β-1,4 linkage), residual toxin activity was tested by well test on the sensitive *S. cerevisiae* strain α’1 (Figure 7C). Unbound toxin forms clear lysis zones around the well. The competitive inhibition of the action of viral protein demonstrated that the β-1,6-glucan exclusively present in pustulan provides binding sites for the K66 toxin. K66 toxin activity was completely abolished by pustulan only.

None of the other polysaccharides tested exhibited K66 protein binding effects, forming lysis zones equal to that of control sample without polysaccharide added. The in vitro and in vivo approaches used here to access the binding specificity suggest that type β-1,6-glucans can play the role of primary cell surface receptor for the K66 toxin.

## 4. Discussion

Our work for the first time provides deep insight into the composition and functioning of the *S. paradoxus* viral killer system. The study stemmed from the comprehensive analysis of the viral sequences, analyzed the genetic factors of the host and targets of the virus-encoded killer toxin.

The high similarity between *S. paradoxus* L-A viruses has been reported recently [6]. This observation is in line with the high relatedness observed previously between *S. paradoxus* dsRNA virus-possessing strains based on the distance matrix of PCR profiles with the microsatellite primer (GTG)_5_ [61]. Here, we demonstrate that L-A viruses from *S. paradoxus* discovered so far are significantly more homogenous than those from *S. cerevisiae.* Within *S. paradoxus* L-A viruses, two clades can be confidently separated: LA-28 type, including SpV-LA-66, SpV-LA-21 and SpV-LA-28, and the rest of *S. paradoxus* L-A viruses, except for the SpV-LA-45.

No sequence homology between different *Saccharomyces sensu stricto* yeast dsRNA M viruses is detected, except for the high similarity of *S. paradoxus* SpV-M66 and SpV-M21 viruses. Even though SpV-LA-66 and SpV-LA-28 are highly related, no homology between SpV-M66 and SpV-M28 in nucleotide or in amino acid level can be documented. Genome organization of SpV-M66 resembles all so far described *S. cerevisiae* and *S. paradoxus* dsRNA M viruses [19,28,62]. The coding region is located at the 5′ terminus, followed by an A-rich sequence and non-coding region with secondary stem-loop structure important for encapsidation at the 3′ terminus. The ORF encoding for a K66 preprotoxin features three potential recognition sites of Kex2 protease, acting in the late Golgi compartment. Thus, the maturation process of K66 toxin may proceed by two alternative scenarios: first, by removing pre-pro-sequence and potential γ-peptide (from 182 till 239 aa) and forming a disulfide-bonded α/β heterodimer. A similar structural organization is typical for almost all known *S. cerevisiae* killer toxins, except for the K2 killer protein lacking γ-subunit [15,62]. In the second scenario, the Kex2 protease may not cleave at the position 239 and, similar to the K2 killer protein, γ-peptide is not released. In this case, the β subunit will start from the amino acid position 182, retaining the integral DUF5341 domain. Future studies are needed to fully understand the role of DUF5341 in the killing phenotype of K66, as this domain is found in numerous proteins of *Ascomycota*, including the N-terminal part of KHS killer toxin. Of special interest is the presence of the DUF5341 domain identified within C-terminal part of the K2 toxin [63]. However, the patterns of DUF5341 sequence similarity to K66 and K2 do not match, making direct sequence comparison not possible. Therefore, K66 and K2 toxins appear to share the same DUF5341 domain core. Three transmembrane helixes detected in the K66 predict this protein to form an ion channel after reaching the plasma membrane, similar to *S. cerevisiae* K2 and K1 toxins [15,25]. The possibility of the K66 toxin acting in monomeric form could not be excluded, as the probability level of disulfide bond prediction is rather low. ER retention motif, typical for K28 protein and essential for its activity [37] was not found in the structure of K66, separating the organization and therefore the modes of action of these toxins further on.

BLAST search revealed K66 homologues in different yeast strains. We found that all ORFs coding homologues of K66 in *S. cerevisiae* are at the telomeric region of chromosome 5 and code for YER187W-like proteins. In some strains, YER187W ORF contains an in-frame stop codon (for example, in *S. cerevisiae* BY4741). YER187W ORF neighboring YER188W is homologous to Kbarr-1 killer toxin (GenBank KT429819), encoded by *Torulaspora delbrueckii* dsRNA Mbarr-1 killer virus. An identity of some regions of *T. delbrueckii* Mbarr-1 genome with the putative replication and packaging signals of most of the M-virus RNAs was observed, suggesting the evolutionary relationship [7]. Several chromosomal ORFs with homology to *S. cerevisiae* Klus, K1, or K2 preprotoxins have been observed in yeasts before, suggesting that the M virus might have originated from the host messenger RNAs, been encapsidated and replicated by the L-A virus-encoded RNA polymerase after acquiring sequences needed for both events, probably from the genome of the L-A virus itself [14]. In addition, it has been demonstrated that genes of dsRNA viruses from *Totiviridae* and *Partitiviridae* have widespread homologues in the nuclear genomes of eukaryotic organisms, such as plants, arthropods, fungi, nematodes, and protozoa, suggesting that viral genes might have been transferred horizontally from viral to eukaryotic genomes [8,64].

Despite the striking similarity in virus genome organization and toxin maturation, the modes of action of the viral toxins are clearly distinct [15]. There are differences between toxins with respect to killing, interaction with the host cells, and immunity formation mechanisms. Mycovirus-originated killer proteins are usually active between pH 4.0 and 5.4 and at a temperature below 30 °C [1,65]. The favorable conditions for the establishment of *Saccharomyces* killer species have been found on fruits where the pH is moderately low [49,66,67], growing best in a natural environment with optimal temperature range of 20 °C to 30 °C [57]. These conditions strongly correlate with activity and stability profile of most secreted killer toxins and are in line with our data pointing to the optimal activity of *S. paradoxus* K66 toxin at a temperature of 20 °C at pH about 4.8. The *S. cerevisiae* killer toxins have narrow target range, inhibiting only strains or species within the same genus [68], except for the Klus toxin, which executes the activity against broader spectrum of yeast [28]. *S. paradoxus* killer strain AML-15-66 exhibits similar features towards the majority of *S. cerevisiae* yeast, demonstrating killing activity against *S. cerevisiae* cells but being not active against other yeast genera tested. The AML-15-66 strain possessed the lowest activity against *S. cerevisiae* K2 toxin-producing strain M437, suggesting relation of both toxins, while K28-bearing *S. cerevisiae* strain M300 was killed the most efficiently among all killer strains tested. At the same time, AML-15-66 was sensitive to the action of *S. cerevisiae* K2 toxin, probably due to the high activity of this toxin [69,70], and completely resistant to other known killer types of *S. cerevisiae* K1, K28, and Klus.

The genetic screen performed on *S. cerevisiae* single-gene deletion strains revealed 125 gene products important for the functioning of the *S. paradoxus* K66 toxin and involved in the target cell susceptibility. Previous yeast genome-wide screens performed with all known dsRNA virus-originated *S. cerevisiae* killer toxins revealed a 753 gene set, contributing to the functioning of viral agents: 268 for the K1, 332 for the K2, and 365 for the K28 [29,30,31]. Page and colleagues [29] determined that the resistance to the *S. cerevisiae* K1 toxin is mostly conferred by gene products linked to the synthesis of cell wall components, secretion pathway, and cell surface signal transduction. Mutant cells with an increased amount of β-1,6-glucans in the cell wall, those unable to grow at high osmolarity, and bearing compromised stress response pathways, exhibited increased sensitivity to the K1 toxin [29]. The K2 toxin executed a similar mechanism of action as K1; therefore, changes in the cell wall structure and proper functioning of mitochondria remain crucial for the killing phenotype. The cells defective in HOG and CWI signaling pathways, with affected maintenance of pH and ion homeostasis, demonstrated hypersensitivity to the K2 toxin [31]. Even for the action of the different-by-mechanism K28 toxin, interrupted cell wall biogenesis process and lipid organization led to increased resistance, while most genes related to hypersensitivity to K28 toxin are those involved in stress-activated signaling and protein degradation [30]. In this study, based on manual annotation, the largest groups of K66 modulators have been identified as those connected to cell wall organization and biogenesis, membrane formation and transport, chromatin organization, and gene expression. GO analysis and protein interconnection networks highlighted the importance of cell wall structure for the functioning of *S. paradoxus* K66 toxin and allowed us to speculate that K66 acts via the disruption of ion homeostasis, since genes involved in regulation of osmotic stress response were highly represented in the screen. Importantly, 85% of modulators identified in the *S. paradoxus* K66 screen (106 gene products) were involved in the functioning of *S. cerevisiae* K1 or K2 toxins as well, pointing to a high similarity of their action. At the same time, only 19 unique modulators common for both *S. paradoxus* K66 and *S. cerevisiae* K28 were found. Functions of several of those gene products are connected to endocytosis and, therefore, remain unclear in the context of *S. paradoxus* K66 toxin predicted function. Until the relevant experiments are carried out, the possibility that the K66 toxin possesses yet another unique mode of the action cannot be excluded.

Yeast cell wall is a primary target for cytotoxic activity of most mycotoxins, and different components of the cell wall could play the role of receptors [71]. Mutants with altered cell wall biogenesis process demonstrated the most resistant phenotype to the action of *S. paradoxus* viral protein K66. This is expected, because the toxin primarily interacts with the cell wall and components of the plasma membrane. By investigating the efficiency of the K66 toxin binding to *S. cerevisiae* mutant cells with altered levels of β-1,3 and β-1,6 glucans in vivo and performing competition experiments with the polysaccharides, bearing different glucan linkages, we observed a good correlation between the level of β-1,6 glucans and binding ability. This highlights the potential of β-1,6 glucans to act as primary targets on the cell wall. The similar genetic factors related to cell wall organization and biogenesis processes modulate the functioning of *S. cerevisiae* K1 and K2 toxins [29,31]. Even more, β-1,6-glucan was originally proposed to be a cell wall receptor for *S. cerevisiae* K1 and K2 toxins [21,22]. Therefore, we propose that β-1,6 glucans could play the role of primary receptors of *S. paradoxus* K66 toxin, further relating its action to that of *S. cerevisiae* K1 and K2 toxins.

Our finding that the majority of L-A viruses from *S. paradoxus* are significantly more homogenous than those from *S. cerevisiae*, might substantiate an important evolutionary crossroad between wild *S. paradoxus* and domesticated *S. cerevisiae* yeast. It remains to be discovered the reason or driving force for the extreme level of homology of L-A viruses from *S. paradoxus*, if any: genomes of *S. paradoxus* were shown to be at least several times more diverse than *S. cerevisiae* genomes [72]. One can envision the evolutionary pressure from the satellite M dsRNA virus; however, many of *S. paradoxus* killer systems are rather diverse in terms of toxin specificity [9,19]. At the same time, *S. paradoxus* SpV-LA-45 is more similar to *S. cerevisiae* counterparts, than to L-As from *S. paradoxus*, extending further limits of L-A virus variability in host species. *S. cerevisiae* features at least four killer systems, maintained by corresponding L-A variants [15,37], therefore highlighting a route for possible diversification of L-A viruses within species. Here, fitness pressure determined by the domestication of *S. cerevisiae* should not be overlooked.

Ample specific combinations of *S. paradoxus* killer systems cast doubts on homogeneity within killer-helper duos of *S. paradoxus*. The specificity paradigm in maintenance of M satellite virus by corresponding L-A virus only has been challenged by the *S. cerevisiae* K2 killer system, where two distinct L-A type viruses were found to support the ScV-M2 virus in wild strains [14,28]. This paradigm is further refused by the *S. paradoxus* K66 killer system, consisting of the previously unreported combination of LA-28 type SpV-LA-66 virus and M1/M2 type SpV-M66 virus. To the best of our knowledge, this is the first report of LA-28 type helper virus maintaining other than the M28 type satellite virus in a wild-type strain. The presented uniqueness of the K66 killer system raises questions of the true limits and the factors behind helper-satellite compatibility and distribution. Genetic background is essential for the consolidating of any virus in a cell; the diversity of yeast killer systems should obey the cellular context, unique for each and every host species. Results from this study extend our knowledge on the *Totiviridae* viruses in *Saccharomyces sensu stricto* yeasts and functioning of killer systems, urging exploration of new horizons of their diversity.

## Figures and Tables

**Table 1:** Killing phenotype of *S. paradoxus* AML-15-66 strain. Diameter of zone of inhibition in mm: +++ (3–2.5), +++/- (2.5–2), ++ (2–1.5), ++/- (1.5–1), + (1–0.5), +/- (0.5–0).

Target Strain (Killer Type)	Killing Phenotype of *S. Paradoxus* AML-15-66							
	pH							
	3.2	3.6	4.0	4.4	4.8	5.2	5.6	6.0
*S. cerevisiae* ἀ 1 (K0)	-	+/-	+	++	+++	++/-	-	-
*S. cerevisiae* BY4741 (K0)	-	+/-	+	++	+++/-	+	-	-
*S. cerevisiae* K7 (K1)	-	-	+/-	+	+	+/-	-	-
*S. cerevisiae* K7 [L-M-] (K0)	-	-	+/-	+	++/-	+	-	-
*S. cerevisiae* M437 (K2)	-	-	+/-	+/-	+/-	+/-	-	-
*S. cerevisiae* M437 [L-M-] (K0)	-	-	-	+/-	+	+/-	-	-
*S. cerevisiae* CRB-15-4 (Klus)	-	-	-	+/-	+	+	+/-	-
*S. cerevisiae* CRB-15-4 [L-M-] (K0)	-	-	-	+/-	+	+	+/-	-
*S. cerevisiae* MS300 (K28)	-	-	+	+	++	+	-	-
*S. cerevisiae* MS300 [L-M-] (K0)	-	-	+	++/-	++	++/-	+/-	-
*S. paradoxus* AML-15-66 (K66)	-	-	-	-	-	-	-	-
*S. paradoxus* AML-15-66 [L-M-] (K0)	-	-	+/-	+	++/-	+/-	-	-
*S. paradoxus* T.21.4 (K21)	-	-	-	-	-	-	-	-

## Supplementary Materials

The following are available online at http://www.mdpi.com/1999-4915/10/10/564/s1. Figure S1: Identification of *S. paradoxus* AML-15-66 strain, Figure S2: Characterization of killing and immunity features of K66 toxin-producing *S. paradoxus* strain, Figure S3: Electrophoretic analysis of dsRNAs extracted from different killer yeast, Figure S4: The similarities of *S. cerevisiae* and *S. paradoxus* dsRNA L-A virus-encoded Gag proteins, Figure S5: Sequence alignment of SpV-M66 and SpV-M21 genomes, Figure S6: Nucleotide sequence of the SpV-M66 genome and amino acid sequence of the putative ORF of K66 toxin, Table S1: Mutant strains with an altered *S. paradoxus* K66 killer toxin phenotype, Table S2: GO terms in biological process for genes involved in the functioning of *S. paradoxus* K66 toxin.
