# Supplementary material for: Saccharomyces paradoxus K66 Killer System Evidences Expanded Assortment of Helper and Satellite Viruses
Source: Viruses. 2018 Oct 16;10(10):564. doi: 10.3390/v10100564 (PMC6213463; doi:10.3390/v10100564)
Supplement: Supplementary file 1 [file viruses-10-00564-s001.zip › Supplemental Figures.pdf]

Supplementary Materials: *Saccharomyces paradoxus* K66 killer system evidences expanded assortment of helper and satellite viruses

Iglė Vepštaitė-Monstavičė, Juliana Lukša, Aleksandras Konovalovas, Dovilė Ežerskytė, Ramunė Stanevičienė, Živilė Strazdaitė-Žielienė, Saulius Serva and Elena Servienė

A

```
Sp AML-15-66 16  AATGGAtttttttGTTTTGGCAGGAGCATGAGAGCTTTTACTGGGCAAGAAGACAAGAGA 75
                 ||||||||||||||||||||||||||||||||||||||||||||||||||||||||||||
Sp CBS:5829   60  AATGGATTTTTTTGTTTTGGCAGGAGCATGAGAGCTTTTACTGGGCAAGAAGACAAGAGA 119

Sp AML-15-66 76  TGGAGAGTCCAGTCGGGCCTGCGCTTAAGTGCGCGGTCTTGCTTGGCTTGTAAGTTTCTT 135
                 ||||||||||||||||||||||||||||||||||||||||||||||||||||||||||||
Sp CBS:5829  120  TGGAGAGTCCAGTCGGGCCTGCGCTTAAGTGCGCGGTCTTGCTTGGCTTGTAAGTTTCTT 179

Sp AML-15-66 136 TCTTGCTATTCCAAACGGTGAGAGATTTCTGTGCTATTGTTATAGGACAATTAAAACCGT 195
                 ||||||||||||||||||||||||||||||||||||||||||||||||||||||||||||
Sp CBS:5829  180  TCTTGCTATTCCAAACGGTGAGAGATTTCTGTGCTATTGTTATAGGACAATTAAAACCGT 239

Sp AML-15-66 196 TTCAATACAACACACTGTGGAGTTTTCATATCTTTGCAACTTTTTCTTTGGGCATTCGAG 255
                 ||||||||||||||||||||||||||||||||||||||||||||||||||||||||||||
Sp CBS:5829  240  TTCAATACAACACACTGTGGAGTTTTCATATCTTTGCAACTTTTTCTTTGGGCATTCGAG 299

Sp AML-15-66 256 CAATCGAGGCCCAGAGGTAACAAACACAAACAATTTTATTTATTCATTAAATTTTTGTCA 315
                 ||||||||||||||||||||||||||||||||||||||||||||||||||||||||||||
Sp CBS:5829  300  CAATCGAGGCCCAGAGGTAACAAACACAAACAATTTTATTTATTCATTAAATTTTTGTCA 359

Sp AML-15-66 316 AAAACAAGAATTTTCGTAACTGGAAATTTTAAAAATATTAAAAACTTTCAACAACGGATC 375
                 ||||||||||||||||||||||||||||||||||||||||||||||||||||||||||||
Sp CBS:5829  360  AAAACAAGAATTTTCGTAACTGGAAATTTTAAAAATATTAAAAACTTTCAACAACGGATC 419

Sp AML-15-66 376 TCTTGGTTCTCGCATCGATGAAGAACGCAGCGAAATGCGATACGTAATGTGAATTGCAGA 435
                 ||||||||||||||||||||||||||||||||||||||||||||||||||||||||||||
Sp CBS:5829  420  TCTTGGTTCTCGCATCGATGAAGAACGCAGCGAAATGCGATACGTAATGTGAATTGCAGA 479

Sp AML-15-66 436 ATTCCGTGAATCATCGAATCTTTGAACGCACATTGCGCCCCTTGGTATTCCAGGGGGCAT 495
                 ||||||||||||||||||||||||||||||||||||||||||||||||||||||||||||
Sp CBS:5829  480  ATTCCGTGAATCATCGAATCTTTGAACGCACATTGCGCCCCTTGGTATTCCAGGGGGCAT 539

Sp AML-15-66 496 GCCTGTTTGAGCGTCATTTCCTTCTCAAACATTCTGTTTGGTAGTGAGTGATACTCTTTG 555
                 ||||||||||||||||||||||||||||||||||||||||||||||||||||||||||||
Sp CBS:5829  540  GCCTGTTTGAGCGTCATTTCCTTCTCAAACATTCTGTTTGGTAGTGAGTGATACTCTTTG 599

Sp AML-15-66 556 GAGTTAACTTGAAATTGCTGGCCTTTTCATTGGATGttttttttCCAAAGAGAGGTTTCT 615
                 ||||||||||||||||||||||||||||||||||||||||||||||||||||||||||||
Sp CBS:5829  600  GAGTTAACTTGAAATTGCTGGCCTTTTCATTGGATGTTTTTTTTCCAAAGAGAGGTTTCT 659

Sp AML-15-66 616 CTGCGTGCTTGAGGTATAATGCAAGTACGGTCGTTTTAGGTTTTACCAACTGCGGCTAAT 675
                 ||||||||||||||||||||||||||||||||||||||||||||||||||||||||||||
Sp CBS:5829  660  CTGCGTGCTTGAGGTATAATGCAAGTACGGTCGTTTTAGGTTTTACCAACTGCGGCTAAT 719

Sp AML-15-66 676 CTTTTTTGTACTGAGCGTATTGGAACGTTATCGATAAGAAGAGAGCGTCTAGGCGAACAA 735
                 ||||||||||||||||||||||||||||||||||||||||||||||||||||||||||||
Sp CBS:5829  720  CTTTTTTGTACTGAGCGTATTGGAACGTTATCGATAAGAAGAGAGCGTCTAGGCGAACAA 779

Sp AML-15-66 736 TGTTCTTAAAGTTTGACCTCAAATCAGGTAGGAGTACCCGCTGAACTTAAGCATATCA   793
                 ||||||||||||||||||||||||||||||||||||||||||||||||||||||||||
Sp CBS:5829  780  TGTTCTTAAAGTTTGACCTCAAATCAGGTAGGAGTACCCGCTGAACTTAAGCATATCA   837
```

B

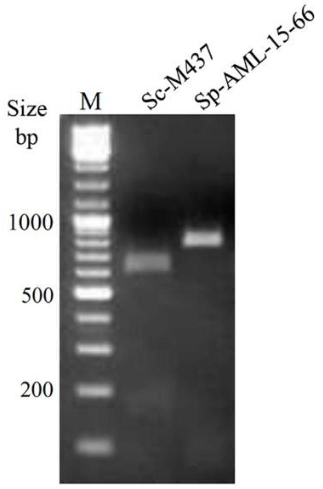

Figure S1. Identification of S. *paradoxus* AML-15-66 strain. A. Similarity of AML-15-66 to *Saccharomyces paradoxus* culture CBS:5829 based on sequences of internal transcribed spacer 1 and 2 including 5.8S ribosomal RNA gene. B. RFLP-PCR profiles of ITS regions of *S. paradoxus* AML-15-66 and *S. cerevisiae* M437 yeasts. FspBI-digested PCR products loaded on 2% agarose gel. M: molecular weight (kb) marker GeneRuler DNA Ladder Mix (Thermo Fisher).

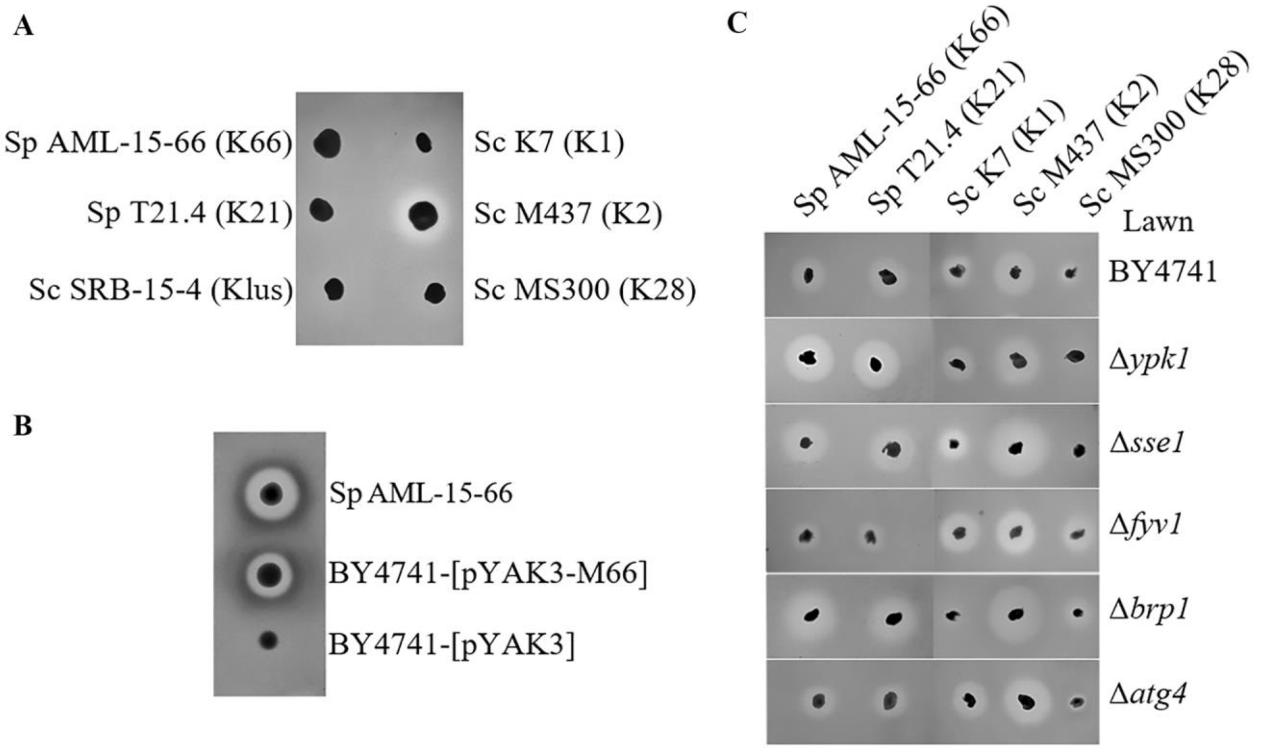

Figure S2. Characterization of killing and immunity features of K66 toxin-producing *S. paradoxus* strain. A. Susceptibility of *S. paradoxus* AML-15-66 strain to the *S. cerevisiae* killer toxins. *S. cerevisiae* strain M437 producing K2 type viral toxin inhibits growth of *S. paradoxus* AML-15-66 cells seeded into MBA agar. Other toxin producing strains did not demonstrate lysis zones on overlay of AML-15-66 cells. B. Killing phenotype of *S. paradoxus* K66 toxin producing *wt* strain and *S. cerevisiae* transformant BY4741-[pYAK3-M66] featuring expressed K66 coding sequence. Plate is seeded with a lawn of the *S. cerevisiae* strain BY4741. C. Representative examples of yeast mutant susceptibility to different killer toxins. The assay was performed by depositing toxin producing cells on the surface of an MBA plate (pH 4.8) inoculated with a mutant strain to test.

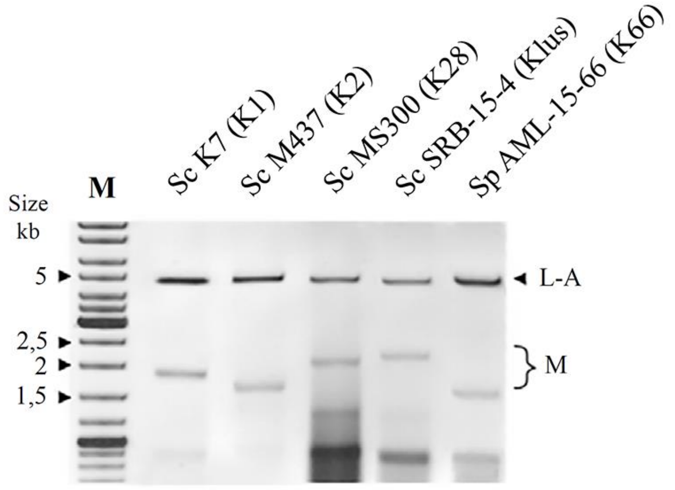

Figure S3. Electrophoretic analysis of dsRNAs extracted from different killer yeast. L-A and M on the right marks type of dsRNA. Source strain and killer type indicated above the picture, M denotes molecular weight marker GeneRuler DNA Ladder Mix (Thermo Fisher Scientific).

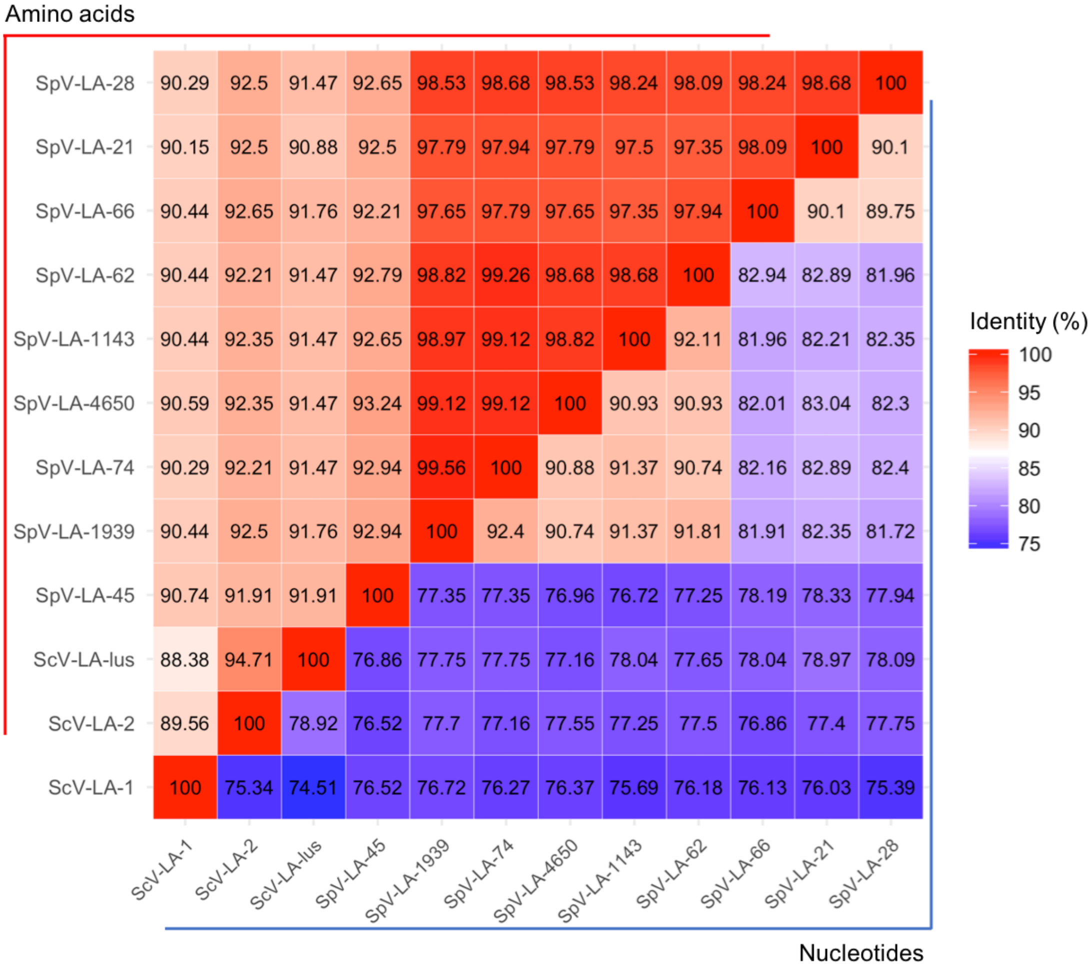

Figure S4. The similarities of *S. cerevisiae* and *S. paradoxus* dsRNA L-A virus-encoded Gag proteins. Identity at nucleotide level is represented in the lower right triangle, amino acid level – in the upper left triangle, framed by corresponding blue and red lines.

```
        1        10        20        30        40        50
SpV-M66 ..GAAAATGTCTAAGCTGTATAATACCTCCAGGGAAGTTACATGGCGTAGCATTTTTTCA
SpV-M21 GAAAAAATGTCTAAGCTGTATAACACCTCTAGGGAAGTTACATGGCGTAGTATTTTTTCA
        60        70        80        90       100       110
SpV-M66 CTTGCCTTTAAGATCATGCTGGTAATGTTCTGTGTCTTAGCTGTATTAACACTGTTAGTA
SpV-M21 CTTGCCCTTAAGATCATGCTGGTAATGTTCTGTGTTTTAGCTGTATTAACGCTGTTAGTG
        120       130       140       150       160       170
SpV-M66 AGCCGGCTTTTAGGCACATCACAATCTGTGGATGTAGAACACTTGAGTATGCTGCATAAA
SpV-M21 AGCCGACTTTTAGGCACATCACAACCCATGGATGTGGAACACTTGAGTAGGCTGCATAAA
        180       190       200       210       220       230
SpV-M66 CGAGCCAGATGGACATGGGTTCAAGGTGCGACTTATGCAGGGCTGCTGTTAGCAGCAGGT
SpV-M21 CGAGCTAGATGGACATGGGTTCAAGGCGCAACTTATGCAGGGCTGTTATTAGCAGCAGGT
        240       250       260       270       280       290
SpV-M66 GCTCTCATAGCTCCGTCGTGGTCAGCAGCAATCTGCCTGGCGACGGTGAAAGACTATTGT
SpV-M21 GCTCTCATAGCTCCGTCTTGGTCAGCAGCAATATGCTTGGCGACGGCGAAGGACTACTGC
        300       310       320       330       340       350
SpV-M66 GCGCCACTAGTTAATGCTATTCTTAGCACGATTGTTGTCAGCGTAGCCGGAGGGATGGCC
SpV-M21 GCACCACTAGCTAATGCTATTCTTAGCACGATTGTTGTCAGCATAGCCGGAGGGATAGCC
        360       370       380       390       400       410
SpV-M66 TGGCGTACTAGCGGTGTGGATATACACCAGCGTGCGCTGCAATCATCGCTGGTTCTGGGC
SpV-M21 TGGCGTACTAGCGGTGTGGGTGTACACGAACGTGCACTGCAATCATCGCTGATTCTCGGC
        420       430       440       450       460       470
SpV-M66 TCTCTCAATCTAACACTCAACGCCGACTTTAACAGTAGTCAACTAGCGGCGGTTGATACC
SpV-M21 TCCCTTAATCTAACACTCAACGCTGACTTCAACAGCAGTCAGCTAGCGGCTGTTGATACC
        480       490       500       510       520       530
SpV-M66 CACCCTCATTTGGTCTCGCTGGATGCATGGACAGTGGGGCCAACGGACTTACACAGATTA
SpV-M21 CACCCGCATTTGGTCTCGTTGGATGCGTGGACAGTGGGGCCAGCAGACCTACGCAGATTA
        540       550       560       570       580       590
SpV-M66 GGTAAACGTGACGAGGATACAGGCAGTTCTGTCAACAACGGCACTTACTTATTCTTCACA
SpV-M21 GGTAAACGTGATGGGGATACAGGTAATTCTGTTAATAACGGCACTTATTTGTTCTTCACG
        600       610       620       630       640       650
SpV-M66 TCCGAATATGGTACTCATACTGCGCACATGCCAGGTGATGATGTTGGTACTCTAGTAGAT
SpV-M21 TCCGAATATGGCACTCATACTGCTCACATGTCAGGTGATGATGTCGGTACTCTAGTAGAC
        660       670       680       690       700       710
SpV-M66 TTTGTATTGAACGCAGTTCCAGACGAGCCTAACATCAATACTACAATTAATTTTACTAAG
SpV-M21 TTTGTGTTAAACGCGATTCCAGACGAGCCTAACATTAATAGCACAATTAATTCTGCTAAG
        720       730       740       750       760       770
SpV-M66 CGGAGTCAGCAATTCGGAGTATCATGGGTGTCATATATATGGACGAAGCCAACCATGAT
SpV-M21 CGGAGTCAACAATTCGGAGTATCATGGGTGTCATATATATGGACGAAGCCAACCATGAT
        780       790       800       810       820       830
SpV-M66 CTGGATACCGAGTGGTATAATGAAGAGGGTGGTAGCTTTGACTCTCAGTTAGAAGAAAGT
SpV-M21 CTGGATACCGAGTGGTATAATGAAGAAGGTGGTAGCTTCGACTCTCAGTTAGAAGAAGGT
        840       850       860       870       880       890
SpV-M66 TTGGCGCAAGCAATAATTGATATACCTGACTGGAAATACTGCATTGCACCAGAAGTCAGT
SpV-M21 TTGACGCAAGCAATAGTTGATATACCTGACTGGAAGTACTGTATTGCACCTGAAGTCAGT
        900       910       920       930       940       950
SpV-M66 AAGGGCGAGGCAATATCTTATGACGACATACCCGGCACGCATGACGGAAATGGTAATGCG
SpV-M21 AAGGGCGAGGCAATATCTTATGACGACATACCCGGCACGCAAAACGGAAATGGCAATGCG
```

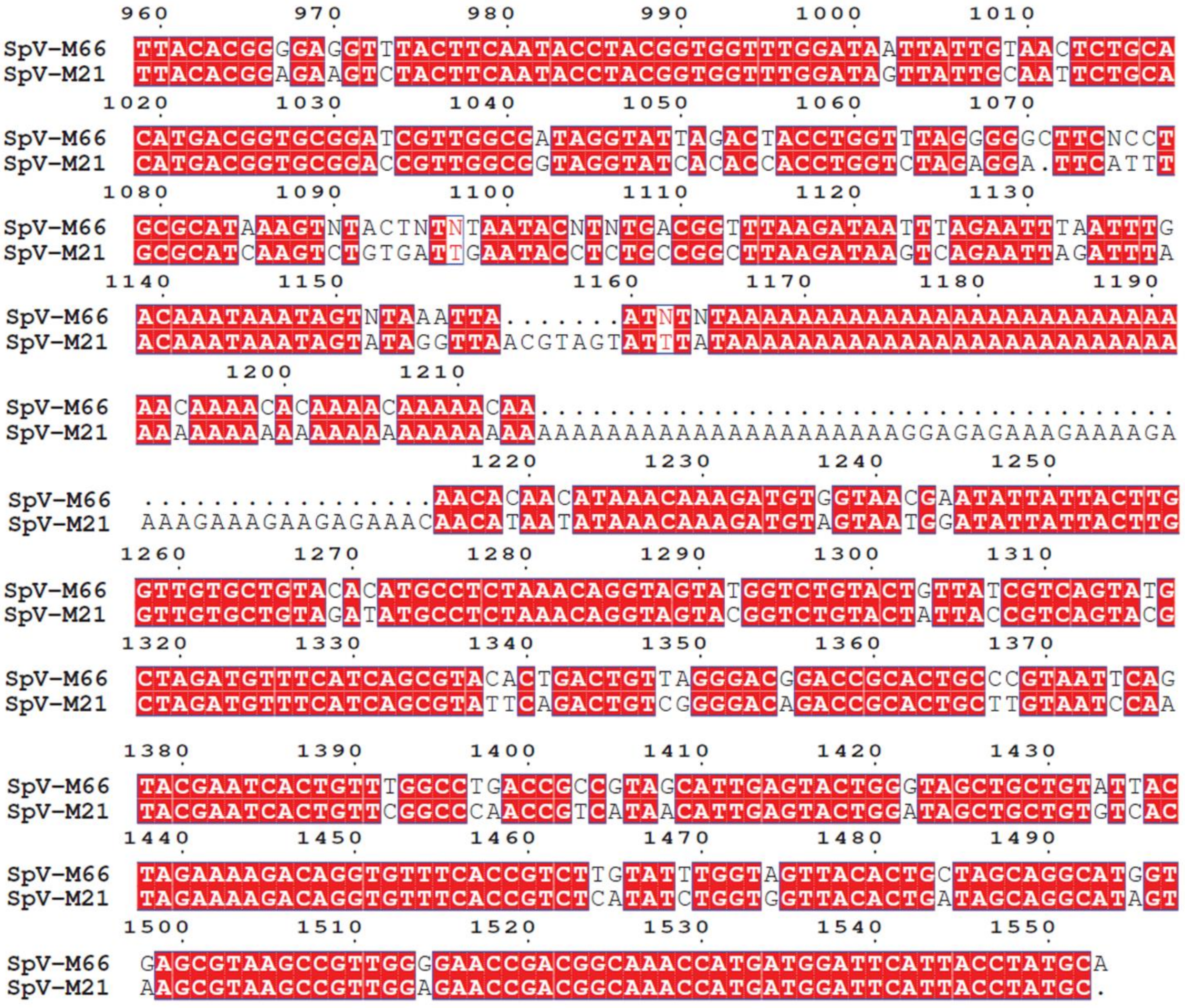

Figure S5. Sequence alignment of SpV-M66 and SpV-M21 genomes.

SpV-M66 (1553 bp)

gaaaatgtctaagctgtataatacctccagggaagttacatggcgtagcatttttcacttgcctttaagatcatgctggtaa
M S K L Y N T S R E V T W R S I F S L A F K I M L V
K66
10 20 30 40 50 60 70 80

tgttctgtgtcttagctgtattaacactgttagtaagccggcttttaggcacatcacaatctgtggatgtagaacacttgagt
M F C V L A V L T L L V S R L L G T S Q S V D V E H L S
K66
90 100 110 120 130 140 150 160

atgctgcataaacgagccagatggacatgggttcaaggtgcgacttatgcagggctgctgttagcagcaggtgctctcatagc
M L H K R A R W T W V Q G A T Y A G L L L A A G A L I A
K66
170 180 190 200 210 220 230 240

tccgtcgtggtcagcagcaatctgcctggcgacggtgaaagactattgtgcgccactagttaatgctattcttagcacgattg
P S W S A A I C L A T V K D Y C A P L V N A I L S T I
K66
250 260 270 280 290 300 310 320 330

ttgtcagcgtagccggagggatggcctggcgtactagcggtgtggatatacaccagcgtgcgctgcaatcatcgctggttctg
V V S V A G G M A W R T S G V D I H Q R A L Q S S L V L
K66
340 350 360 370 380 390 400 410

ggctctctcaatctaacactcaacgccgactttaacagtagtcaactagcggcggttgatacccaccctcatttggtctcgct
G S L N L T L N A D F N S S Q L A A V D T H P H L V S L
K66
420 430 440 450 460 470 480 490

ggatgcatggacagtggggccaacggacttacacagattaggtaaacgtgacgaggatacaggcagttctgtcaacaacggca
D A W T V G P T D L H R L G K R D E D T G S S V N N G
K66
500 510 520 530 540 550 560 570 580

cttacttattcttcacatccgaatatggtactcatactgcgcacatgccaggtgatgatgttggtactctagtagatttttgta
T Y L F F T S E Y G T H T A H M P G D D V G T L V D F V
K66
590 600 610 620 630 640 650 660

ttgaacgcagttccagacgagcctaacatcaatactacaattaattttactaagcggagtcagcaattcggagtatcatgggt
L N A V P D E P N I N T T I N F T K R S Q Q F G V S W V
K66
670 680 690 700 710 720 730 740

gtcatatatatgggacgaagccaaccatgatctggataccgagtggtataatgaagagggtggtagctttgactctcagttag
S Y I W D E A N H D L D T E W Y N E E G G S F D S Q L
K66
750 760 770 780 790 800 810 820 830

aagaaagtttggcgcaagcaataattgatatacctgactggaaatactgcattgcaccagaagtcagtaagggcgaggcaata
E E S L A Q A I I D I P D W K Y C I A P E V S K G E A I
K66
840 850 860 870 880 890 900 910

tcttatgacgacatacccggcacgcatgacggaaatggtaatgcgttacacggggaggtttacttcaatacctacggtggttt
S Y D D I P G T H D G N G N A L H G E V Y F N T Y G G L
K66
920 930 940 950 960 970 980 990

ggataattattgtaactctgcacatgacggtgcggatcgttggcgataggtattagactacctggtttagggggcttcncctg
D N Y C N S A H D G A D R W R
K66
1,000 1,010 1,020 1,030 1,040 1,050 1,060 1,070

cgcataaagtntactntntaatacntntgacggtttaagataatttagaatttaatttgacaaataaatagtntaaattaatn
1,080 1,090 1,100 1,110 1,120 1,130 1,140 1,150 1,160

tntaaaaaaaaaaaaaaaaaaaaaaaaaacaaaacacaaaacaaaaacaaaacacaacataaacaaagatgtggtaacgaa
polyA
3'-UTR
1,170 1,180 1,190 1,200 1,210 1,220 1,230 1,240

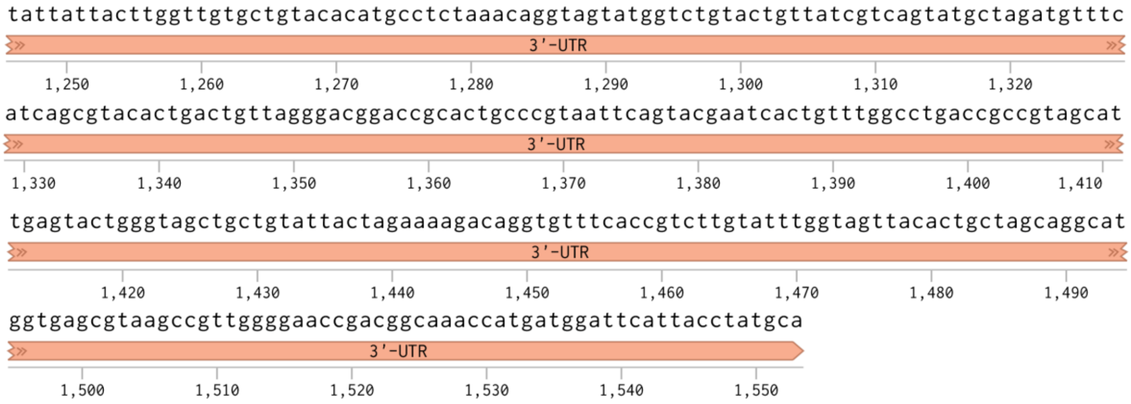

Figure S6. Nucleotide sequence of the SpV-M66 genome and amino acid sequence of the putative ORF of K66 toxin.
